# Physical activity effects on bladder dysfunction in an obese and insulin‐resistant murine model

**DOI:** 10.14814/phy2.14792

**Published:** 2021-04-27

**Authors:** André Matos de Oliveira, Fernando Mello Froes Fonseca, Sabrina Thalita Reis, Nayara Izabel Viana, Edilamar Menezes Oliveira, Luiz Osório Leiria, Katia Ramos Moreira Leite, William Carlos Nahas, Miguel Srougi, Alberto Azoubel Antunes

**Affiliations:** ^1^ Laboratory of Medical Research – LIM 55, Urology University of Sao Paulo Medical School Sao Paulo Brazil; ^2^ School of Physical Education and Sport University of Sao Paulo São Paulo Brazil; ^3^ Department of Pharmacology Faculty of Medical Sciences State University of Campinas Campinas Brazil

**Keywords:** bladder, nitric oxide, obesity, overactive bladder, physical activity

## Abstract

**Objective:**

To investigate the role of physical activity in functional and molecular bladder alterations in an obese and insulin‐resistant murine model.

**Methods:**

Wistar rats were randomized into 1. physical activity and standard diet; 2. physical activity and high‐fat diet; 3. no physical activity and standard diet; and 4. no physical activity and high‐fat diet. Groups 1 and 2 were subjected to a 10‐week swimming protocol. Urodynamic study (UDS) was performed, and the expression of genes in the bladder tissue related to the insulin pathway (IRS1/IRS2/PI3K/AKT/eNOS) was assessed using quantitative real‐time polymerase chain reaction.

**Results:**

Groups 1 and 2 presented lower body weight gains than groups 3 (213.89 ± 13.77 vs 261.63 ± 34.20 grams (g), *p* = 0.04) and 4 (209.84 ± 27.40 vs 257.57 ± 32.95 g, *p* = 0.04), respectively. Group 4 had higher insulin level (6.05 ± 1.79 vs 4.14 ± 1.14 ng/ml, *p* = 0.038) and higher homeostasis model assessment of insulin resistance (HOMA‐IR) index (1.95 ± 0.73 vs 1.09 ± 0.37, *p* = 0.006) than group 1. On UDS, group 4 had greater number of micturition (13.6 ± 4.21 vs 6.0 ± 1.82, *p* = 0.04), higher postvoid pressure (8.06 ± 2.24 vs 5.08 ± 1.23, *p* = 0.04), lower capacity (0.29 ± 0.18 vs 0.91 ± 0.41 ml, *p* = 0.008), and lower bladder compliance (0.027 ± 0.014 vs 0.091 ± 0.034 ml/mmHg, *p* = 0.016) versus group 1. High‐fat diet was related to an underexpression throughout insulin signaling pathway, and physical activity was related to an overexpression of the pathway.

**Conclusions:**

The insulin signaling pathway may be involved in the pathogenesis of bladder dysfunction related to a high‐fat diet. Physical activity may help to prevent bladder disfunction induced by a high‐fat diet through the insulin pathway.

## Graphical abstract

We investigated the role of physical activity in functional and molecular bladder alterations in an obese and insulin‐resistant murine model. The insulin signaling pathway IRS2/PI3K/AKT/eNOS may be involved in the pathogenesis of bladder dysfunction related to a high‐fat diet. Physical activity may help to prevent bladder disfunction induced by a high‐fat diet through the insulin pathway.

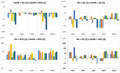

## 1 INTRODUCTION

Lower urinary tract symptoms (LUTS) represent an important public health problem because of their high prevalence and deleterious effects. A large‐scale epidemiological study in the United States, England, and Sweden (EpiLUTS) found an incidence of at least one LUTS (except incontinence) reported as a frequency threshold of “often” (a few times per week) in 47.9% and 52.5% of male and female patients, respectively (Coyne et al., 2009). The etiology of LUTS is multifactorial, and recent evidence suggests an association between LUTS and factors outside the urinary tract such as chronic diseases (heart disease and diabetes) and lifestyle (diet and physical activity) (Fitzgerald et al., 2007).

The World Health Organization estimates that approximately 1.3 in each 10 adults are obese (Jaime et al., 2013). Human obesity is strongly influenced by hypercaloric diets combined with low energy expenditure (sedentary lifestyle) (Galgani & Ravussin, 2008). A study with obese rats fed with a high‐fat diet (HFD) for 24 weeks showed an increase in voiding and nonvoiding contractions, suggesting bladder hyperactivity.(Rahman et al., 2007) Obesity is also believed to be the main etiological factor in peripheral tissue insulin resistance (Kahn & Flier, 2000).

Insulin is involved with vascular relaxation through mechanisms involving the nitric oxide, which is also formed in the urothelial cells. One of the mechanisms through which insulin acts in bladder tissue has recently been discovered; insulin activates the PI3K/AKT/eNOS pathway in the mucosa, with a consequent release of NO to the detrusor muscle, resulting in muscle relaxation (Scherrer et al., 1994; Winder et al., 2014). Obese and insulin‐resistant mice exhibited inadequate insulin action on the bladder mucosa, with a reduced tissue eNOS concentration and increased frequency of detrusor contractions (Leiria et al., 2013).

A recent meta‐analysis revealed that individuals who are physically active have a lower risk of developing LUTS (Parsons & Kashefi, 2008). However, the exact mechanism through which physical activity may improve LUTS is still unknown. Additionally, the effects of physical activity on the bladder insulin pathway described above have not been studied so far. The objective of the present study is to evaluate the role of physical activity in functional and molecular bladder changes in the PI3K/AKT/eNOS pathway in obese and insulin‐resistant rats.

## 2 METHODS

### 2.1 Animals and swimming training protocol

Twenty‐eight male Wistar rats (8 weeks) were housed 3–5 per cage with free access to chow and water. The study was conducted according to the ethical principles for animal research adopted by the Brazilian College of Animal Experimentation. (Ethics board and animal approved project number: 374/12). Rats were randomly assigned to four groups: 1. physical activity (PA) and standard diet (SD); 2. PA and HFD; 3. no physical activity (NoPA) and SD; and 4. NoPA and HFD.

The SD (AIN 93M – Pragsoluções Biociências Ltda) was prepared with 62% starch, 14% protein, 10% sucrose, and 4% soy oil. The HFD (Pragsoluções Biociências Ltda) was composed of 36% starch, 14% protein, 30% fat (lard), 10% sucrose, and 4% soy oil.

The training sessions consisted of 60‐minute swimming five days/week for 10 weeks in a pool containing warmed water (32°C). A week before the beginning of the protocol, we adapted the rats on swimming progressively, adding 10 minutes a day. After six days, the rats were able to swim for 60 minutes. This swimming protocol has been characterized previously as low to moderate intensity and long duration. (Barretti et al., 2012; Medeiros et al., 2004).

### 2.2 Tail‐cuff blood pressure measurements

Blood pressure (BP) was measured during the 12‐week study using a computerized tail‐cuff system (Kent Scientific, Torrington, CT, USA). A cuff was placed around tail, and systolic pressure was recorded (AT/CODAS, 100‐Hz sampling rate, DataQ Instruments, Inc., Akron, OH, USA).

### 2.3 Urodynamic study

Rats were anesthetized with an intraperitoneal injection of urethane (1.2 g kg−1). A 1‐cm abdominal incision was made, and a butterfly cannula (23 G) was inserted into the bladder dome. The cannula was connected to a three‐way tap, one port of which was connected to a pressure transducer (Stoelting Co. Wooddale, Illinois, USA) and the other to an infusion pump (Samtronic Infusion Pump 670). Initially, the bladder was emptied. Continuous cystometry was carried out by infusing saline into the bladder at a rate of 5 ml/h. After first spontaneous micturition, parameters were assessed for 30 minutes. The following parameters were assessed: frequency of voiding contractions (nmic), intercontraction interval (ICI), threshold pressure (Pthres)—the intravesical pressure immediately before micturition; postvoid pressure (PVP)—the intravesical pressure immediately after micturition; peak pressure (Pmax)—the peak pressure reached during micturition; capacity (Cap)—the volume of saline needed to induce the first micturition; and compliance (Bcompl)—the ratio of Cap to Pthres, the volume required for a unit rise of pressure measured during filling.

### 2.4 Serum and molecular tissue measures

At the end of cystometry and still under anesthesia, rats were decapitated, and blood was collected from cervical vessels. Blood samples were collected, centrifuged, and stored at −80°C. A blind investigator performed ELISA method in order to quantified serum glucose, total cholesterol and fraction, triglyceride, and insulin levels. The homeostasis model assessment of insulin resistance (HOMA‐IR) index was calculated as follows: HOMA‐IR = serum insulin x serum glucose/22.5. All animals were in 8 hours fasting period.

Bladders were extracted and stored at −80°C. After that, we extracted and weighted epididymal and retroperitoneal fat. Both are related to visceral obesity in rats. The relative gene expression of insulin receptor substrate 1 and 2 (IRS1 and IRS2, respectively), phosphoinositide‐3‐kinase (PI3K), protein kinase B (AKT), and endothelial nitric oxide synthase (eNOS) were assessed using quantitative real‐time polymerase chain reaction (RT‐PCR).

### 2.5 Isolation of RNA and cDNA synthesis

For RNA extraction, we used a mirVana kit (Ambion, Austin, TX), and cDNA was obtained using a high‐capacity cDNA reverse transcription kit. A quantity of 10 ng of RNA was subjected to reverse transcription using a random primer. The reaction for obtaining cDNA was performed with a Veriti Thermal Cycler (Applied Biosystems, Foster City, CA) for 10 min at 25°C, 120 minutes at 37°C, and 5 minutes at 85°C.

### 2.6 Analysis of gene expression

The expression levels of the RNA were analyzed using an ABI 7500 Fast RT‐PCR System (Applied Biosystems). Target sequences were amplified in a 10‐ml reaction vessel containing 5 ml of TaqMan Universal PCR Master Mix, 0.5 ml of TaqMan Gene Expression Assays (primers: Supporting Information), 1 ml of cDNA, and 3.5 ml of DNase‐free water. The polymerase chain reaction cycling conditions were 2 minutes at 50°C, 10 minutes at 95°C, and 40 cycles of 15 seconds at 95°C and 1 minute at 60°C. All reactions were performed in duplicate. A TaqMan beta‐2‐microglobulin (B2M; Applied Biosystems, Foster City, CA) assay was utilized as endogenous control. We used the delta–delta cycle threshold (DDCT) method to calculate the relative expression of genes using the equation DDCT = (CT target gene, PA rat sample −CT endogenous control, PA rat sample) − (CT target gene, NoPA rat sample −CT endogenous control, NoPA rat sample). The fold change in gene expression was calculated as 2‐∆∆CT (Livak & Schmittgen, 2001).

### 2.7 Statistical analysis

Quantitative variables are summarized as the mean and standard deviation. Statistical analysis was performed using SPSS 20.0 (IBM, Armonk, NY), using ANOVA for multivariate analysis. (Level of significance of 5%).

## 3 RESULTS

### 3.1 Morphometric characteristics

The weight gain of the rats subjected to physical activity was significantly lower than no physical activity, regardless of the diet (group 1: 213.89 g vs group 3: 261.63 g (*p* = 0.04); group 2: 209.84 g vs group 4: 257.57 g (*p* = 0.04) There was no difference in systolic blood pressure between groups (*p* > 0.05) (Table 1).

**TABLE 1:** Morphometric characteristics of the groups of studied rats (results are expressed as the mean ± standard deviation)

	Group 1 PA + SD (n = 7)	Group 2 PA + HFD (n = 7)	Group 3 NoPA + SD (n = 7)	Group 4 NoPA + HFD (n = 7)
Weight gain (g)	213.89 (±13.77)bc	209.84 (±27.40)de	261.63 (±34.20)	257.57 (±32.95)
Epididymal fat (g)	14.06 (±4.04)bc	15.26 (±4.53)de	19.74 (±4.09)	20.38 (±3.44)
Retroperitoneal fat (g)	15.35 (±5.31)c	16.62 (±6.04)	18.23 (±3.23)	21.58 (±4.53)
Systolic blood pressure (mmHg)	115.44 (±12.98)	113.40 (±13.08)	110.38 (±13.14)	117.57 (±13.93)
Blood glucose (mg/dl)	106.86 (±4.98)ac	123.33 (±19.88)d	104.86 (±5.67)f	129.29 (±16.24)
Insulin (ng/ml)	4.14 (±1.14)c	4.87 (±2.24)	5.14 (±0.78)	6.05 (±1.79)
HOMA‐IR index	1.09 (±0.37)c	1.49 (±0.64)	1.33 (±0.21)f	1.95 (±0.73)
Cholesterol (mg/dl)	63.5 (±3.93)a b	54.95 (±7.08)d	71.74 (±8.30)f	61.34 (±6.98)
LDL (mg/dl)	2.69 (±1.42)	2.75 (±1.76)	4.39 (±2.57)	2.73 (±1.13)
HDL (mg/dl)	46.53 (±3.83)	38.58 (±8.16)	45.08 (±10.02)	45.19 (±8.27)
Triglycerides (mg/dl)	71.43 (±14.49)b	68.08 (±18.49)d	111.32 (±28.04)f	67.11 (±10.70)

Abbreviations: HFD, high‐fat diet; NoPA, no physical activity; PA, physical activity; SD, standard diet.

(a) *p* < 0.05 for the group 1 × 2 comparison; (b) *p* < 0.05 for the group 1 × 3 comparison; (c) *p* < 0.05 for the group 1 × 4 comparison; (d) *p* < 0.05 for the group 2 × 3 comparison; (e) *p* < 0.05 for the group 2 × 4 comparison; and (f) *p* < 0.05 for the group 3 × 4 comparison.

In both groups fed with HFD, the serum glucose was higher (group 1: 106.86 mg/dl vs group 2, 123.33 mg/dl (*p* = 0.03), group 3: 104.86 mg/dl vs group 4: 129.29 mg/dl (*p* = 0.02). The serum insulin was higher in group 4 than group 1 (6.05 ng/ml vs 4.14 ng/ml, *p* = 0.038). Similarly, HOMA‐IR index was higher in group 4 (1.95) and was significantly higher than that in both groups fed a SD (group 1: 1.09, *p* = 0.006 and group 3, 1.33, *p* = 0.038) (Table 1).

### 3.2 Urodynamic data

Group 4 (SED +HFD) had a significantly higher nmic (13.60 ± 4.21 vs 6.00 ± 1.82, *p* = 0.04), higher PVP (8.06 ± 2.24 vs 5.08 ± 1.23 mmHg, *p* = 0.04), lower Cap (0.29 ± 0.18 vs 0.91 ± 0.41 ml, *p* = 0.008), and lower Bcompl (0.027 ± 0.014 vs 0.091 ± 0.034 ml/mmHg, *p* = 0.016) than group 1 (PA + SD) (Table 2). There was no significant difference between the groups in ICI and Pmax (Table 2).

**TABLE 2:** Urodynamic parameters of the groups of rats studied (results are expressed as the mean ±standard deviation)

	Group 1 PA +SD (n = 4)	Group 2 PA +HFD (n = 5)	Group 3 NoPA +SD (n = 7)	Group 4 NoPA +HFD (n = 5)
Number of micturitions (nmic)	6.00 (±1.82)c	10.80 (±4.81)	8.43 (±3.55)	13.60 (±4.21)
Intercontraction interval (ICI)	228.92 (±77.84)	184.80 (±93.63)	185.34 (±56.38)	132.18 (±42.03)
Threshold pressure (Pthres) (mmHg)	9.00 (±1.24)	8.93 (±1.12)	8.00 (±1.03)f	10.96 (±2.08)
Peak pressure (Pmax) (mmHg)	25.94 (±3.85)	31.06 (±3.78)	28.53 (±1.77)	29.40 (±6.12)
Postvoid pressure (PVP) (mmHg)	5.08 (±1.23)c	6.53 (±1.52)	4.79 (±0.87)f	8.06 (±2.24)
Capacity (Cap) (ml)	0.91 (±0.41)ac	0.37 (±0.14)	0.59 (±0.22)	0.29 (±0.18)
Compliance (Bcompl) (ml/mmHg)	0.091 (±0.034)c	0.039 (±0.14)	0.073 (±0.036)	0.027 (±0.014)

Abbreviations: HFD, high‐fat diet; NoPA, no physical activity; PA, physical activity; SD, standard diet.

(a) *p* < 0.05 for the group 1 × 2 comparison; (b) *p* < 0.05 for the group 1 × 3 comparison; (c) *p* < 0.05 for the group 1 × 4 comparison; (d) *p* < 0.05 for the group 2 × 3 comparison; (e) *p* < 0.05 for the group 2 × 4 comparison; and (f) *p* < 0.05 for the group 3 × 4 comparison

### 3.3 Molecular expression

**FIGURE 1:**
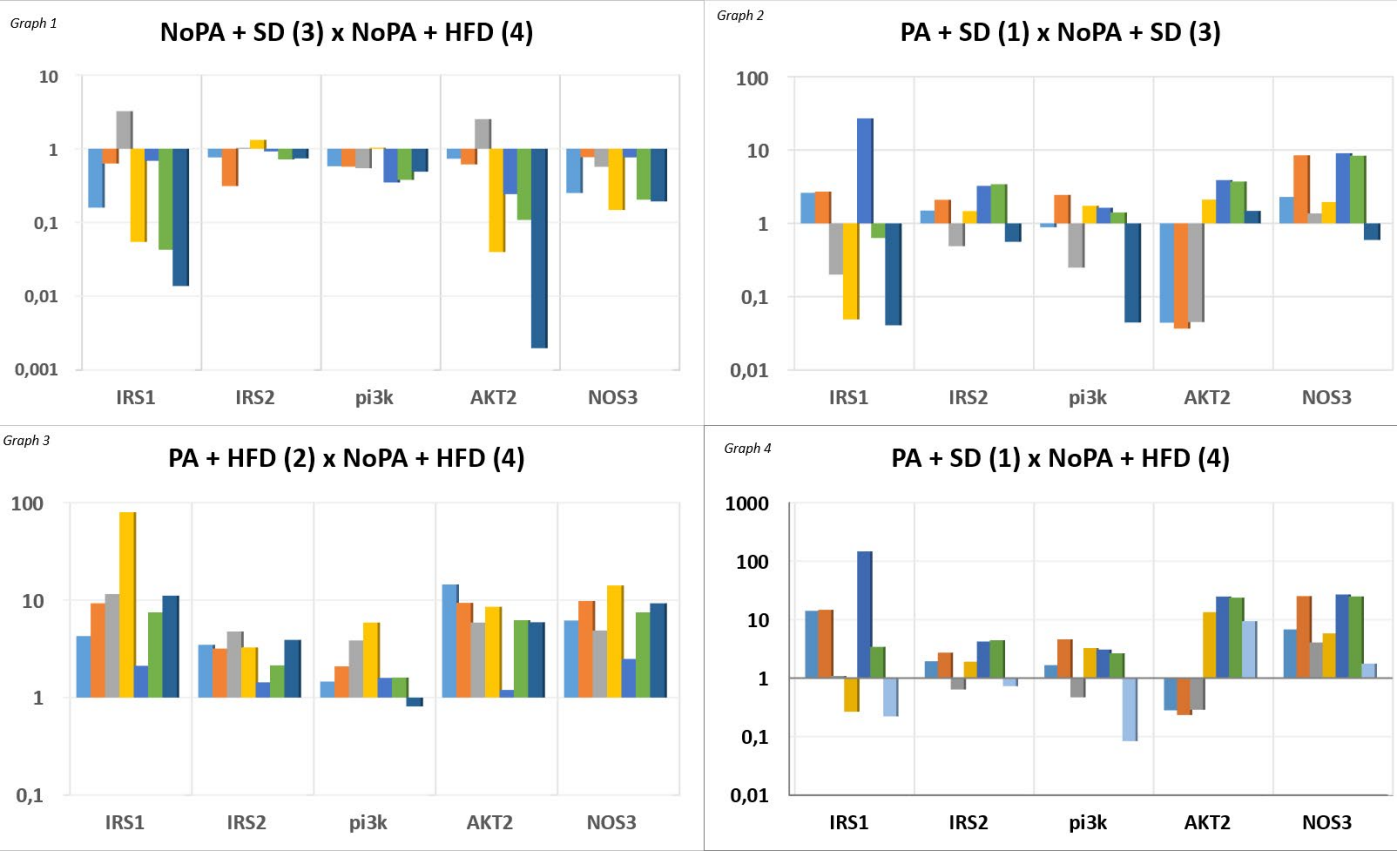
Ratio of relative gene expression for each rat in comparison with the mean of control group (baseline). Graph 1: No physical activity groups (baseline: group 3); Graph 2: standard diet groups (bseline: group 3); Graph 3: high‐fat diet groups (baseline: group 4); and Graph 4: “extreme” groups (baseline: group 4). Legend: NoPA: no physical activity; SD: standard diet; HFD: high‐fat diet; PA: physical activity; IRS1: insulin receptor substrate‐1; IRS2: insulin receptor substrate‐2; PI3K: phosphoinositide‐3‐kinase; AKT2: protein kinase B; NOS3: endothelial nitric oxide synthase

The effect of the HFD on the molecular expression (IRS1, IRS2, PI3K, AKT, and NOS3) was analyzed by comparing no physical activity rats fed a SD (group 3) and a HFD (group 4). There was lower expression of all genes in the latter group (Figure 1/Graph 1; Table 3).

**TABLE 3:** Relative gene expressions in comparison with NoPA fed a HFD (4) and NoPA fed a SD (3) (control), PA (1) and NoPA (3) (control) groups of rats fed a SD, PA (2) and NoPA (4) (control) groups of rats fed a HFD, PA fed a SD (1) NoPA rats fed a HFD (4) (control)

Group 4 vs 3	IRS1	IRS2	PI3K	AKT2	NOS3
Mean	**0.68x**	**0.83x**	**0.56x**	**0.61x**	**0.41x**
Median	**0.16x**	**0.76x**	**0.54x**	**0.24x**	**0.25x**
Overexpression	14.3%	28.6%	14.3%	14.3%	0
Subexpression	85.7%	71.4%	85.7%	85.7%	100%

Group 1 vs 3	IRS1	IRS2	PI3K	AKT2	NOS3
Mean	**4.75x**	**1.82x**	**1.20x**	**1.61x**	**4.59x**
Median	**0.63x**	**1.49x**	**1.41x**	**1.47x**	**2.28x**
Overexpression	42.8%	71.4%	42.8%	42.8%	85.7%
Subexpression	57.2%	28.6%	57.2%	57.2%	14.3%

Group 2 vs 4	IRS1	IRS2	PI3K	AKT2	NOS3
Mean	**17.97x**	**3.17x**	**2.47x**	**7.38x**	**7.75x**
Median	**9.28x**	**3.27x**	**1.61x**	**6.22x**	**7.50x**
Overexpression	100%	100%	85.7%	100%	100%
Subexpression	0	0	14.3%	0	0

Group 1 vs 4	IRS1	IRS2	PI3K	AKT2	NOS3
Mean	**25.83x**	**2.37x**	**2.26x**	**10.32x**	**13.65x**
Median	**3.43x**	**1.94x**	**2.65x**	**9.41x**	**6.80x**
Overexpression	71.4%	71.4%	71.4%	57.2%	100%
Subexpression	18.6%	18.6%	18.6%	42.8%	0

The level of expression of the target genes was obtained by the relative quantification and the levels of expression determined by the 2‐ △△ CT method. On the logarithmic graph, this method standardizes the expression of normal control as a baseline (represented by the number 1) and the relative expression of each RNA for each sample is sometimes shown as normal for more in cases of overexpression and for less in cases of underexpression. All reactions were performed in duplicate (in bold) (Livak & Schmittgen, 2001).

Abbreviations: HFD, high‐fat diet; NoPA, no physical activity; PA, physical activity; SD, standard diet.

To evaluate the isolated effect of physical activity on molecular expression, the active and no active rats on the SD were compared (group 1 vs 3 comparison). There was overexpression of IRS2 and NOS3 in active rats (71.4% and 85.7% of the rats, respectively) (Figure 1/Graph 2; Table 3).

The analysis of the effect of physical activity on the molecular expression in rats fed a HFD was performed by comparing the active and no active rats fed the HFD (group 2 vs 4 comparison). There was overexpression in all studied genes, with IRS1, IRS2, AKT2, and NOS3 being overexpressed in 100% of rats (Figure 1/Graph 3; Table 3).

Analysis of the "extreme" groups was performed by comparing the active rats fed a SD with no active rats fed HFD (group 1 vs group 4). There was overexpression of all studied genes in group 1, with NOS3 being overexpressed in 100% of the rats (Figure 1/Graph 4; Table 3).

## 4 DISCUSSION

In the present analysis, we demonstrated that physical activity may influence bladder function and bladder expression of genes related to the insulin pathway in rats with obesity and insulin resistance. Although animals submitted to HFD did not gain more weight than those submitted to standard diet, after 12 weeks the NoPA rats became more obese in comparison with PA rats, regardless of the diet used. Metabolically, when the effects of the two interventions were combined (NoPA and HFD), the rats of group 4 demonstrated a significantly higher mean insulin level and HOMA‐IR index than that of group 1; this metabolic pattern is suggestive of insulin resistance. Using the same comparison (group 1 vs 4), urodynamic analysis showed features suggestive of bladder hyperactivity in group 4—higher nmic and PVP and lower Cap and Bcompl. For the molecular study of the insulin signaling pathway in the bladder tissue, the HFD resulted in subexpression of genes throughout the entire pathway (IRS1, IRS2, PI3K, AKT, and NOS3). In contrast, physical activity resulted in overexpression of genes in the pathway, especially when comparing the rats fed a HFD (group 2 vs 4) and when comparing the extreme groups (group 1 vs 4).

### 4.1 Metabolic characteristics

Obesity results in peripheral tissue insulin resistance, a key aspect in the development of metabolic syndrome (Kahn & Flier, 2000). Regular physical activity has been proposed as a protective factor against overweight and obesity (Donnelly et al., 2009; Kahn & Flier, 2000). Our rats subjected to the physical activity protocol gained significantly less weight, regardless of diet, suggesting that this activity is a protective factor against weight gain in the animals. In a recent study, Yang et al. (2016) also demonstrated the protective effect of physical activity (swimming) on Sprague–Dawley rats on a HFD. After 16 weeks of training, the HFD‐fed rats that underwent swimming training (five times/week) had a significantly lower weight gain than the rats that only consumed a HFD (without swimming).

The benefits of physical activity on blood glucose metabolism, such as better control of serum glucose and greater sensitivity of peripheral tissues to insulin, have been reported in obese patients with type 2 *diabetes mellitus*, suggesting a positive correlation between body weight loss and better blood glucose control (Cuff et al., 2003). However, physical activity alone without body weight reduction may not provide benefits for blood glucose metabolism, suggesting an important role of caloric intake (diet) in the weight reduction process. In 2002, Poirier et al. evaluated the effect of a 12‐week aerobic exercise program on insulin sensitivity in obese and non‐obese type 2 diabetic patients. At the end of the intervention period, non‐obese patients had improved insulin sensitivity, which did not occur in obese patients who did not lose weight (Poirier et al., 2002).

Regarding the lipid profile, the total cholesterol and triglyceride levels were highest in group 3 (NoPA and SD) and were significantly higher than those in all the other groups. However, the cholesterol fractions (HDL and LDL) did not significantly differ between the groups. This is an unexpected result for us, and probably, a longer period of diet (6 months) and/or a greater concentration of fat could provide a hyperlipidemia pattern. Oberbach et al exposed Sprague–Dawley rats to a high‐fat diet with 45% of saturate diet, during a 11‐week period and found a hyperlipidemia pattern (Oberbach et al.,^2013^).

### 4.2 Functional characteristics

When analyzing the extreme groups (1 vs 4), group 4 exhibited a higher nmic (*p* = 0.04), higher PVP (*p* = 0.04), lower Cap (*p* = 0.008), and lower Bcompl (*p* = 0.016), which is indicative of bladder hyperactivity. Rahman et al. (2007) in 2007, used a 10% saturated fat (lard) diet for 6 months in Sprague–Dawley rats, leading to obese rats with erectile dysfunction and bladder hyperactivity. In 2013, Leiria et al. (2013) demonstrated bladder hyperactivity in a model with C57BL6/J mice fed a HFD (55% fat) for 10 weeks. The obese animals had a higher number of bladder contractions, with or without voiding. Our HFD rat groups were fed with a 30% lard diet for 12 weeks. In the literature, only one experimental study correlates physical activity with bladder functional behavior in the context of diabetes. In 2011, Vadhavkar et al. (2011) studied female diabetic mice (db/db) subjected to a running program during 8 weeks. After intervention, they performed cystometry study of the animals and showed a reduction in nmic and a reduction in residual volume in the diabetic rats who were physically active, suggesting improvement of bladder function (Vadhavkar et al., 2011).

### 4.3 Molecular characteristics

In 1992, Persson et al. (1992) used cystometry in rats and demonstrated that inhibition of the L‐arginine/nitric oxide pathway resulted in bladder hyperactivity and reduced bladder capacity. In 2008, Persson et al. (1992) studied an animal model with NO deficiency secondary to the use of L‐NAME and found increased detrusor muscle contractility in response to muscarinic agonists. Zecchin et al demonstrated the effect of the HFD on this signaling pathway (Zecchin et al., 2007). When studying the aortic tissue of rats fed a HFD, they found impairment in the IRS1/PI3K/AKT/eNOS signaling pathway in response to insulin, with a consequent impairment of local vasodilation. In our study, there was subexpression of all genes (IRS1, IRS2, PI3K, AKT, and NOS3) in the NoPA rats fed a HFD, with NOS3 being underexpressed in 100% of the cases. (median ‐ 0.25x). These results suggest the deleterious effect of the HFD on tissue sensitivity to insulin, with reduced expression of eNOS and thus a likely decrease in NO production.

Wang et al. (2015) used a myocardial infarction model in Sprague–Dawley rats to study the effect of 8 weeks of physical activity on the PI3K/AKT/eNOS signaling pathway in cardiac muscle. Considering a direct relationship between myocardial infarction and insulin resistance, physical activity reestablished local signaling, normalizing the tissue expression of eNOS independent from the blood glucose metabolism (Wang et al., 2015). Silva et al. (2016) studied an obesity model induced by a HFD in mice and the effect of physical activity (treadmill running) on eNOS expression in vascular tissue. After 8 weeks, physical exercise induced a significant increase in eNOS expression in the aorta, improving the vascular functional impairment caused by obesity (Silva et al., 2016).

In 2013, Leiria et al demonstrated the insulin relaxation effects in human and mouse bladder via activation of the PI3K/AKT/eNOS pathway in the bladder mucosa (Leiria et al., 2013). Our study is the first in the literature to describe the protective effect of physical activity on the insulin signaling pathway in bladder tissue, reinforcing the well‐established recommendations for lifestyle changes as an adjuvant in the treatment of bladder dysfunction/LUTS secondary to metabolic alterations.

The present study does have some limitations. Our obesity/insulin resistance model was induced by a HFD of 30% saturated fat for 3 months. A longer period of exposure to the diet and/or a higher concentration of fat may reinforce the metabolic results. To assess insulin resistance, we used the HOMA‐IR index. The gold standard for analyzing this parameter is the hyperinsulinemic–euglycemic clamp test (DeFronzo et al., 1979). However, its use has several limitations, such as high cost, the need for an infusion pump and the long application time. Regarding the functional analysis of the bladder, the urodynamic analysis of anesthetized rats provides a sample study and is possibly less appropriate than a study with conscious animals. Finally, we studied only one tissue signaling pathway, and we understand that the complex network of molecular events resulting from insulin resistance results in multifactorial causes of bladder dysfunction. Future studies should be conducted to demonstrate the results of the present study in humans. A prospective and controlled study with a physical activity intervention in obese men could confirm the benefits of this intervention regarding the voiding pattern and quality of life of the patients.

In conclusion, our results suggest that the insulin signaling pathway IRS2/PI3K/AKT/eNOS may be involved in the pathogenesis of bladder dysfunction related to a high‐fat diet. Physical activity may help to prevent bladder disfunction induced by a high‐fat diet through the insulin pathway. Further clinical studies are necessary to reinforce this hypothesis.

## CONFLICTS OF INTEREST

None.

## AUTHOR CONTRIBUTIONS

AMO and FMFF collected data, analyzed and interpreted data, and critically revised manuscript. STR, NIV, EMO, and LOL analyzed and interpreted data. KRML, WCN, MS, and AAA contributed to study design and critically revised the manuscript.

## Supporting information



Supplementary Material
