# Supplementary material for: Physical activity effects on bladder dysfunction in an obese and insulin‐resistant murine model
Source: Physiol Rep. 2021 Apr 27;9(7):e14792. doi: 10.14814/phy2.14792 (PMC8077148; doi:10.14814/phy2.14792)
Supplement: Supplementary file 1 — Supplementary Material [file PHY2-9-e14792-s001.docx]

**Supporting information**

*Primers* used for quantifying the selected genes.

| Primer | Assay |
| --- | --- |
| IRS1 | Rn02132493_s1 |
| Akt | Rn00690900_m1 |
| PI3K | Rn00564547_m1 |
| IRS2 | Rn01482270_s1 |
| NOS3 | Rn02132634_s1 |
| B2M | Rn560865_m1 |
